# The calcitonin gene-related peptide receptor antagonist MK-8825 decreases spinal trigeminal activity during nitroglycerin infusion

**DOI:** 10.1186/1129-2377-14-93

**Published:** 2013-11-20

**Authors:** Stephan Feistel, Stephanie Albrecht, Karl Messlinger

**Affiliations:** 1 Institute of Physiology and Pathophysiology, University of Erlangen-Nürnberg, Universitätsstr 17, 91054 Erlangen, Germany

**Keywords:** CGRP receptor antagonist, Nitroglycerin, Trigeminal nucleus caudalis, Extracellular recording, Headache, Migraine

## Abstract

**Background:**

Calcitonin gene-related peptide (CGRP) and nitric oxide (NO) are regarded as key mediators in migraine and other primary headaches. Migraineurs respond to infusion of nitroglycerin with delayed headaches, and inhibition of CGRP receptors has been shown to be effective in migraine therapy. In animal experiments nitrovasodilators like nitroglycerin induced increases in spinal trigeminal activity, which were reversed after inhibition of CGRP receptors. In the present study we asked if CGRP receptor inhibition can also prevent spinal trigeminal activity induced by nitroglycerin.

**Methods:**

In isoflurane anaesthetised rats extracellular recordings were made from neurons in the spinal trigeminal nucleus with meningeal afferent input. The non-peptide CGRP receptor inhibitor MK-8825 (5 mg/kg) dissolved in acidic saline (pH 3.3) was slowly infused into rats one hour prior to prolonged glyceryl trinitrate (nitroglycerin) infusion (250 μg/kg/h for two hours).

**Results:**

After infusion of MK-8825 the activity of spinal trigeminal neurons with meningeal afferent input did not increase under continuous nitroglycerin infusion but decreased two hours later below baseline. In contrast, vehicle infusion followed by nitroglycerin was accompanied by a transient increase in activity.

**Conclusions:**

CGRP receptors may be important in an early phase of nitroglycerin-induced central trigeminal activity. This finding may be relevant for nitroglycerin-induced headaches.

## Background

Calcitonin gene-related peptide (CGRP) and nitric oxide (NO) are regarded as key mediators in the generation of migraine and other primary headaches [1,2], based on several clinical data. CGRP has been found at elevated concentrations in jugular venous blood and saliva during attacks of migraine and cluster headache attacks [3]–[5], though this finding is not without contradiction [6]. Infusion of low doses of CGRP caused delayed migraine-like headaches in a group of migraineurs different to healthy control persons [7], possibly indicating a special sensitivity for this neuropeptide associated with migraine predisposition. Furthermore, migraine and cluster headache attacks can be treated with triptans, which is paralleled by normalisation of CGRP levels [3,8], or by CGRP receptor inhibitors like olcegepant (BIBN 4096BS) and telcagepant (MK-0974) [9]–[11]. Likewise, nitrovasodilators such as nitroglycerin can induce delayed migraine-like headaches in migraineurs [12] and other types of headaches in primary headache patients [13,14]. Blockade of the endogenous NO generation with an unspecific NO synthase inhibitor was successful in alleviating migraine pain [15], while selective inhibitors of the inducible NO synthase seemed to be ineffective in reducing or preventing migraine pain [16,17]. From these clinical data it can be concluded that endogenous CGRP and NO, most likely produced from neuronal or endothelial NO synthases, may play a role in the generation of migraine pain and other primary headaches.

The clinical data are paralleled by animal experiments demonstrating the involvement of CGRP and NO in meningeal nociception, which is assumed to underlie the generation of headaches. Particularly, electrophysiological recordings in the rat spinal trigeminal nucleus caudalis from neurons that receive afferent input from meningeal tissues provide a reliable integrative measure of trigeminal activity. Infusion of the nitrovasodilators sodium nitroprusside (SNP) and nitroglycerin (glyceryl trinitrate, GTN) induced ongoing activity of spinal trigeminal neurons with meningeal afferent input [18], resembling the clinical experiments in migraineurs. The increase in activity started after 20–40 min and plateaued after 1–2 hours. Conversely, L-NAME, a non-specific inhibitor of endogenous NO production, reduced the pre-existing spontaneous activity to about the half [19]. Similarly, the CGRP receptor antagonist olcegepant (BIBN4096BS) applied intravenously decreased both spontaneous and heat-evoked spinal trigeminal activity in a dose-dependent manner [20]. Importantly, the activity evoked by SNP and nitroglycerin was also decreased after CGRP receptor blockade to the pre-existing level, suggesting an interaction of CGRP and NO mechanisms in neuronal activity evoked by nitrovasodilators [18].

In the present study we have used MK-8825, a new potent inhibitor for rat CGRP receptors, which showed concentration-dependent inhibition of capsaicin-evoked dermal blood flow with an EC_50_ of about 7.4 μM [21]. MK-8825 is structurally related to the CGRP receptor inhibitor MK-3207, which proved to be effective in the acute treatment of migraine [9,11].

The rationale for the present study was to examine if CGRP receptor blockade by MK-8825 can prevent the increase in neuronal activity induced by nitroglycerin using the aforementioned model of spinal trigeminal recordings. Answering this question may reveal if CGRP receptors are involved in the nitroglycerin-induced promotion of neuronal activity and may have clinical relevance regarding the time of intervention with a CGRP receptor antagonist during the development of migraine attacks.

## Methods

### General procedures

The study was performed in accordance with the ethical guidelines of the International Association for the Study of Pain and the German laws for animal protection and treatment of laboratory animals. The protocol was reviewed by an ethics committee and authorised by the local district government.

Twenty-eight adult male Wistar rats, bred and held in the Institute’s own animal house, with body weights ranging from 250 to 410 g were used. Rats were initially anaesthetised by breathing 4% isoflurane (Forene, Abbott, Wiesbaden, Germany) using an evaporator system (Vapor 19.3, Dräger, Lübeck, Germany). After quick insertion of a tracheal tube they were artificially ventilated (Rodent Ventilator, Ugo Basile, Comerio VA, Italy) with 2% isoflurane in oxygen-enriched room air. Catheters were introduced into the right femoral artery and vein to record systemic blood pressure and to administrate substances. The arterial catheter was permanently perfused with saline (rate 0.2 ml/h) supplemented with 1 IU/ml heparin (Heparin-Natrium-5000-ratiopharm, Ulm, Germany). End-expiratory CO_2_ was monitored (Artema MM 200, Karl Heyer, Bad Ems, Germany) and held at 3% by modulating the ventilation frequency between 70 and 100 strokes per minute, which suppressed spontaneous breathing. Body temperature was measured by a rectal probe and maintained at 37–37.5°C by a feedback controlled heating pad (TKM 0902, Föhr Medical Instruments, Frankfurt, Germany). Arterial pressure was between 70 and 100 mmHg (Pressure Monitor BP-1, World Precision Instruments, Sarasota, Florida). The vital parameters were recorded during the whole experiment. The depth of narcosis during the experiment was constant, so that noxious pinch stimuli did not evoke nociceptive reflexes or changes in arterial blood pressure. At the end of the experiment the animals were euthanised by intravenous injection of an overdose of thiopental (Trapanal, Nycomed, Konstanz, Germany).

### Specific surgery

The animals were transferred to a stereotaxic frame to hold the head in a fixed horizontal position using ear bars and a snout clamp. The skin was cut along the midline from the bregma to the lamda and the skin flaps were retracted laterally. The right parietal bone was carefully trepanised under liquid cooling using a dental drill to expose the dura mater in the cranial window. Care was taken to keep the dura mater intact and to avoid bleeding from dural blood vessels. The dura was protected from drying with 0.9% saline during the experiment. The neck muscles were detached from their insertions, separated in the midline and held apart with a clamp. The medullary brainstem was made accessible by cutting the atlanto-occipital ligament and the spinal dura underneath.

### Recordings

For extracellular recordings of signals a custom-made carbon fibre glass electrode was inserted into the spinal trigeminal nucleus caudalis of the ipsilateral medulla. Using a microstepper, the electrode was moved through the brainstem at steps of 2.5 μm. Single units with meningeal receptive fields were detected by their firing of action-potentials in response to mechanical probing of the parietal dura mater. Signals were filtered, amplified and processed (CED 1401, Cambridge Electronic Design, Cambridge, UK). Spike-analysis was done offline using the discharges generated by mechanical stimulation of meningeal receptive fields as a template (Spike 2 software application, Cambridge Electronic Design).

At the end of the experiments the recording position relative to the obex was assessed by a two-dimensional micrometer system. In some experiments an electric lesion was made by anodal current passed through the recording electrode, and after thiopental injection the animal was perfused in deep anaesthesia through the left ventricle with saline followed by paraformaldehyde solution (4%) for fixation. The brainstem was dissected and histologically processed to localise the recording site.

### Experimental protocols

**Figure 1:**
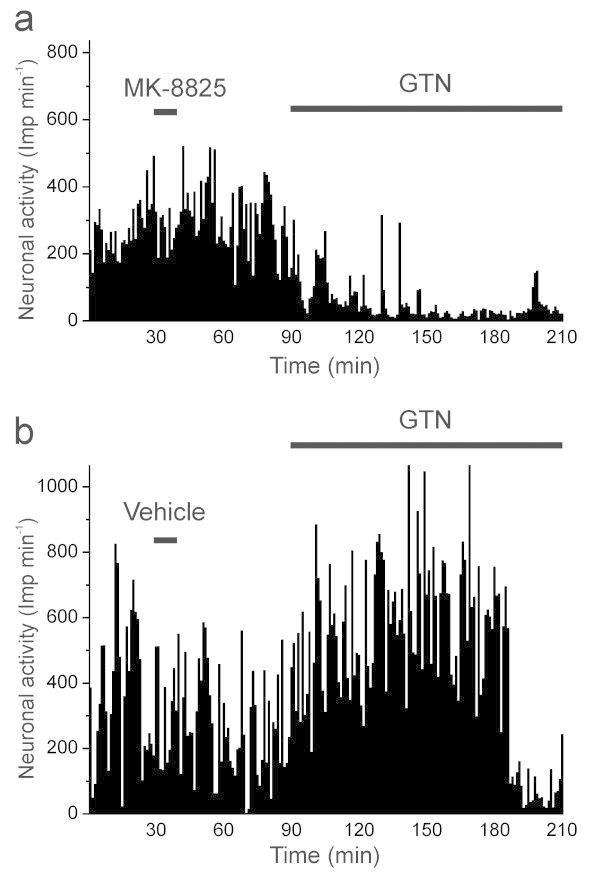
**Histograms showing typical patterns of neuronal activity.** Unit from the sample treated with MK-8825 **(a)** and from the vehicle group **(b).** Vehicle or MK-8825 (5 mg/kg) was slowly i.v. infused between 30 and 40 min, followed by a continuous infusion of nitroglycerin (GTN, 250 μg/kg/h) 60 min later.

When the neuronal activity of a unit was visibly stable, its baseline was recorded for a period of 30 min without any treatment. In 12 experiments the CGRP receptor antagonist MK-8825 (5 mg/kg) dissolved in saline (1 ml/kg body weight, pH 3.3) was i.v. infused at a constant rate within a period of 10 minutes and the recording was continued for further 50 minutes (see Figure 1). In 11 control experiments vehicle (buffered saline, pH 3.3) was infused instead of MK-8825, which was a possible confounding factor. Stimulation with acidic solutions can cause sensitisation of spinal trigeminal neurons with receptive fields in the rodent cranial dura mater [22]. The infusion of MK-8825 or vehicle was followed by a continuous infusion of nitroglycerin (GTN, 250 μg/kg/h) over a period of 120 min. The infusion rate of nitroglycerin was low enough to avoid lowering of systemic blood pressure by more than 5 mmHg, as figured out in previous experiments [18]. In six additional experiments nitroglycerin was infused at the same rate without pre-administration of MK-8825 or vehicle.

### Chemicals

CGRP receptor antagonist MK-8825 (courtesy of the Merck & Co. Inc., NJ, USA) was dissolved in acidic saline (pH 3.3) and i.v. injected at a concentration of 5 mg/kg within 10 minutes. Acidic saline of pH 3.3 was used as vehicle instead of MK-8825. Nitroglycerin (Trinitrosan, Merck Serono, 250 μg/kg/h) was dissolved in 1 ml isotonic saline for i.v. infusion.

### Data analysis and statistics

Statistical tests were performed using Statistica 7.0 software (StatSoft, Tulsa, OK, USA). In the sample of experiments combining MK-8825/vehicle with nitroglycerin (*N* = 12/11) the course of activity was analysed by one- and two-way ANOVA with repeated measurements followed by Fisher’s least significant difference (LSD) post hoc test using regular intervals of 10 and 30 min. The Wilcoxon matched pairs test was used to compare the neuronal activity within periods of 30 min in the group of units treated only with nitroglycerin (*N* = 6, normal distribution not verifiable). Differences were considered significant at *P* < 0.05. For the diagrams the activity was normalised to the mean baseline activity.

## Results

### General properties of neurons

Twenty-nine units recorded in 28 experiments were included in this study. The recording sites were located 1.3 - 3.25 mm caudal to the obex, 0.35 – 1.75 lateral to the midline and at depths of 386 – 1302 μm below the dorsal surface of the medulla. The ongoing activity of these units during the baseline period of 30 min ranged from 7 to 1685 (mean 481) spikes per minute. The meningeal receptive fields of the units were mostly located close to the middle meningeal artery. The mechanical threshold, determined with graded von Frey filaments in the most sensitive center of the receptive fields, ranged from 0.78 to 11.8 mN. The units were activated by electrical square pulses (duration 1 ms) applied to the meningeal receptive fields, thresholds were ranging from 0.4 to 2.4 mA. The latencies after a single electrical pulse close above threshold ranged from 12 to 60 ms. With an assumed conduction distance of 25 mm from the stimulation site to the caudal medulla the recorded units were concluded to be driven by slowly conducting Aδ and/or C-fibres from the dura mater. All units received convergent input from facial areas in the ophthalmic, maxillary or mandibular division of the trigeminal nerve as well as from the temporal muscle or the neck muscles and the periosteum around the cranial window.

### Neuronal responses to MK-8825 followed by nitroglycerin

**Figure 2:**
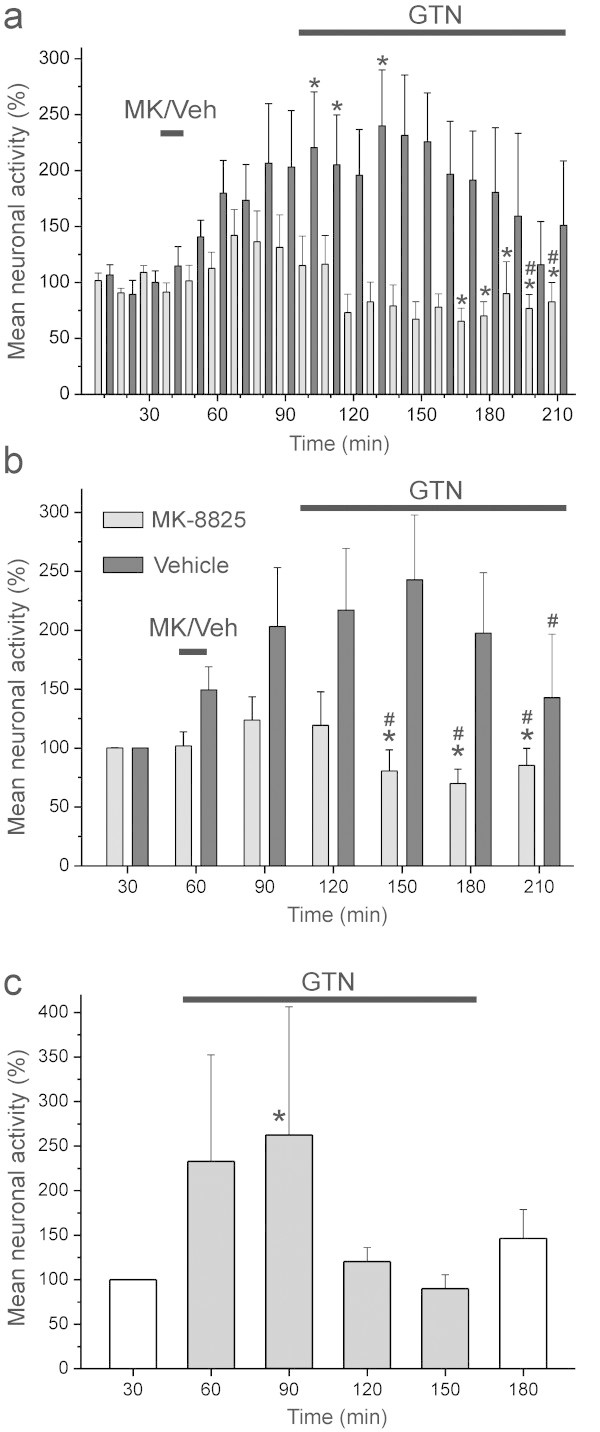
**Histograms showing mean neuronal activity.** Activity was normalised to the baseline of 10 min periods **(a)** and of 30 min periods **(b)** in the MK-8825 group (grey bars, n = 12) and the vehicle group (black bars, n = 11); error bars are s.e.m. **(c)** Mean normalised activity (+ s.e.m.) of 6 units treated with nitroglycerin (GTN) alone for two hours. Significant differences in activity compared to the baseline periods (0–30 min) are indicated by a star and compared to periods after vehicle/MK-8825 infusion by #; repeated measures ANOVA and LSD post hoc test **(a, b)** or WILCOXON **(c)**.

After recording of baseline activity the infusion of MK-8825 (5 mg/kg) was not accompanied by significant changes in activity (Figure 1a), though there was a slight tendency towards a transient increase in the whole sample (see Figure 2a-b). After one hour during the subsequent continuous infusion of nitroglycerin (250 μg/kg/h) the activity decreased below baseline levels in the whole sample of units (*N* = 12, ANOVA, *F*_20,220_ = 2.90, *P* < 0.001). Finally the activity within all 10 min periods during the last 50 min was significantly lower compared to all 10 min periods before infusion of nitroglycerin (LSD test, *P* < 0.05 each; Figure 2a). Comparing periods of 30 min between the MK-8825 and the vehicle sample, two-way ANOVA with repeated measurements indicated a difference in time course (*F*_6,120_ = 2.73, *P* < 0.02), which was due to a significant decrease in activity within the last 90 min of the MK-8825 group compared to baseline and to the period of MK-8825 infusion (LSD test, *P* < 0.05 each; Figure 2b).

### Neuronal responses to vehicle followed by nitroglycerin

The activity of units varied considerably following infusion of vehicle (saline pH 3.3) and nitroglycerin (Figure 1b). Repeated measures ANOVA indicated a significant change in activity during the recording time (*N* = 11, ANOVA, *F*_20,200_ = 1.96, *P* = 0.01). During the infusion of vehicle there was a tendency towards an increase in the whole sample without reaching significance (see Figure 2a-b). The subsequent infusion of nitroglycerin was accompanied by a significant increase in activity (Figure 1b) which in the whole sample of units was significant within the first 20 min and the period of 30–40 min after onset of the nitroglycerin infusion compared to the baseline (LSD test, *P* < 0.05 each; Figure 2a). In the second hour of nitroglycerin infusion the activity declined. Two-way ANOVA with repeated measurements for analysis of 30 min periods in the MK-8825 and the vehicle sample showed that the activity was lowered within the last 30 min of the vehicle group compared to the period of nitroglycerin infusion. (LSD test, *P* < 0.05; Figure 2b).

### Neuronal response to nitroglycerin alone

To re-examine the apparent decrease in activity within the second hour of nitroglycerin infusion, we applied nitroglycerin without any other treatment for two hours in six additional experiments. The activity increased significantly during the second 30 min period after onset of the nitroglycerin infusion (Wilcoxon matched pairs test, p < 0.05) but during the second hour of nitroglycerin infusion it returned to control levels (Figure 2c).

In none of the experiments systemic parameters changed significantly during the infusions.

## Discussion

In the present study we performed extracellular recordings in the caudal spinal trigeminal nucleus from neurons with receptive fields in the cranial dura mater. The activity of these neurons is a good integrative measure of meningeal nociception and is assumed to correlate with headaches in humans [23,24]. Previous experiments have shown that in most of these neurons intravenous infusion of nitrovasodilators like sodium nitroprusside or nitroglycerin induced a delayed or continuous increase in activity, which was reduced to baseline after infusion of the CGRP receptor antagonist olcegepant [18,23]. The main purpose of the present study was to examine if inhibition of CGRP receptors with the new CGRP receptor antagonist MK-8825 is also able to *prevent* an increase in neuronal activity induced by nitroglycerin.

### Study design and effects of nitroglycerin

We have used a continuous low-dose infusion of nitroglycerin to avoid significant lowering of the blood pressure that could occur at higher doses. Since MK-8825 must be dissolved in acidic saline (pH 3.3), the vehicle was titrated to the same pH. The infusion of these acidic solutions was followed by a trend towards an increase in activity in several units without reaching statistical significance in the whole sample of experiments. We assume that the acidic solution was responsible for this tendency, though the acidic solution should have been rapidly buffered in the plasma. Acidic solutions have been shown to induce CGRP release in the hemisected rat head preparation [24] and to activate spinal trigeminal neurons when applied to meningeal receptive fields [25] but there are no data yet with systemic application of acidic substances.

In previous experiments, in which we used the same mode of nitroglycerin infusion, we observed an increase in spinal trigeminal activity that started immediately and reached significance 25 min after onset of the infusion [18]. In the present study we repeated these experiments with a longer observation time, since in the previous study the activity was observed only for one hour. It should be mentioned that the nitroglycerin effect is rather variable between different neurons suggesting that only a fraction of central neurons is readily under the control of NO. Within the first hour of nitroglycerin infusion the activity increased significantly in the whole sample of experiments but the increase did not outlast the second hour of infusion.

Nitroglycerin in clinically relevant concentrations has been shown to cause vasorelaxation not necessarily dependent of NO production, rather by direct activation of the soluble guanylate cyclase (sGC) dependent intracellular pathway [26,27]. Since vasorelaxation induced by nitroglycerin can be independent of CGRP release, it is not surprising that CGRP receptor inhibitors like telcagepant are not effective in inhibiting nitroglycerin induced vasodilation in humans [17]. We assume that the observed transient increase in activity of spinal trigeminal neurons is due to direct sGC activation. Nitroglycerin, though at a considerably higher systemic dose (10 mg/kg) as in the present study, has been shown to activate the transcription factor NF-κB [28] and the calmodulin dependent protein kinase II alpha (CamKIIα) [29] in the rat spinal trigeminal nucleus, which could explain transcriptional effects causing long-term neuronal changes. Therefore it seems likely that nitroglycerin and NO donors cause transient but also transcriptional effects with a delayed onset and long duration. The upregulation of CGRP and nNOS immunoreactivity in rat primary trigeminal afferents six hours after onset of a nitroglycerin infusion at the same dose as in the present study [30] and the increase in NOS-positive second order neurons, which continued during many hours [31,32], are certainly based on long-term transcriptional effects. Glutamate receptors in the spinal trigeminal nucleus seem to be involved in NO-sGC signalling in regard of both immediate neuronal effects [33] and transcriptional effects, which could be attenuated by the unspecific glutamate receptor inhibitor kynurenic acid [34].

Transient receptor potential channels type A1 (TRPA1) have been found to be expressed in peptidergic trigeminal afferents [35,36] and to cause CGRP release followed by meningeal vasodilatation when activated by irritant environmental stimuli [37]. There is also evidence from recent experiments in our laboratory that NO species can induce CGRP release from meningeal afferents via activation of TRPA1 receptor channels, indicating that this pathway may come into account to trigger migraine [38]. This issue should be paid attention to, since TRPA1 can also be activated by volatile anaesthetics [39]. Therefore it was important in our experiments to hold the anaesthesia at a constant level to warrant reproducibility of the measurements.

### Effects of the CGRP receptor inhibitor MK-8825

MK-8825 is related to the compound MK-3207, a CGRP receptor antagonist with high affinity to human but reduced affinity to rat CGRP receptors. MK-8825 has similar potency as MK-3207, but has a much higher unbound fraction in rat plasma. MK-3207 shows inhibition of capsaicin-induced dermal vasodilatation in rhesus monkey [40], and in an adaptive dose-ranging trial MK-3207 demonstrated a positive response on the primary 2-hour pain freedom endpoint and the secondary endpoint of 2-hour pain relief in migraine [41]. Both MK-3207 and MK-8825 are structurally distinct from telcagepant.

After pre-treatment with MK-8825 the infusion of nitroglycerin was not followed by the typical slow increase in neuronal activity. There was rather a tendency towards lowering of activity in the whole sample of experiments, and this decrease was finally significant in the last 90 min of recording. We conclude that this effect was due to the inhibition of CGRP receptors by MK-8825 and that CGRP receptors may be necessary for the action of nitroglycerin to generate neuronal activity.

Time-dependent differential effects of nitroglycerin, as discussed above, could explain the discrepancies between studies using 5-HT1B/D receptor agonists (triptans) to modulate nitroglycerin actions. It has been reported that the increase in nNOS immunoreactivity in the rat spinal trigeminal nucleus observed one hour after 10 mg/kg i.v. nitroglycerin was inhibited by sumatriptan administered shortly before the nitroglycerin infusion [42]. However, in a parallel study with the same systemic (s.c.) nitroglycerin dose and a similar dose of sumatriptan, no inhibition of the increased nNOS expression was found four hours after nitroglycerin [43]. In mice sumatriptan reduced allodynia induced by nitroglycerin, and in cats iontophoretically administered eletriptan normalised the elevated discharge rate of spinal trigeminal neurons after nitroglycerin [44], both acute experiments. Thus it seems that the fast (within one hour) appearing neuronal effects of nitroglycerin can be attenuated by blocking CGRP release or CGRP receptors, while later this is no longer possible. From this we can further conclude that the fast neuronal effects of nitroglycerin depend on the presence of CGRP, while for the long-lasting effects probably based on gene transcription this is doubtful.

### Clinical implications

The slow increase in spinal trigeminal activity induced by nitrovasodilators [23] is paralleled by clinical experiments in healthy persons and patients suffering from primary headaches, in which infusion of nitroglycerin caused delayed headaches [12]–[14]. The data of the present study suggest that inhibition of CGRP receptors could also be effective in *preventing* experimental headaches. In a cross-over study in patients suffering from migraine without aura, in which 13 patients received either olcegepant or placebo after a 20 min infusion of nitroglycerin, migraine developed in 7 patients after olcegepant and in 9 patients after placebo, and the headache scores were similar [45]. The authors concluded that a preventive effect of olcegepant for nitroglycerin-induced migraine pain could not been confirmed. The discrepancy between this study and our experimental data, apart from species differences, may result from differing preconditions between the two study designs. In our study MK-8825 was infused 60 min prior to the continuous nitroglycerin infusion, whereas in the clinical experiments of the above study [45] olcegepant was infused after nitroglycerin. In our study the increase in activity occurred in the first hour and was transient, whereas the delayed headache attacks in the clinical study occurred after several hours. It seems likely that the increase in activity induced by nitroglycerin is a result of the presumed fast effects discussed above, whereas the delayed headaches belong to the long-term events based on gene transcription. Further experiments should clarify if pre-administration of a CGRP receptor inhibitor can also prevent the late events induced by nitroglycerin.

The second question is related to the site of action of CGRP. From animal experiments we know that CGRP applied on rat dura mater is not activating meningeal afferents [46]. Thus, if headache is induced by CGRP, it is most likely a central effect. Assuming that the effects of MK-8825 and olcegepant in meningeal antinociception and migraine therapy, respectively, may result from inhibition of central CGRP receptors, the effects depend essentially from the degree of blood brain penetration of both substances. The question to which extent CGRP receptor antagonists need to target central CGRP receptors for a therapeutic action is controversely discussed [47]–[49]. Animal experiments provide evidence for the involvement of central CGRP receptors to effectively inhibit spinal trigeminal activity [50]. Olcegepant is thought to penetrate the blood brain barrier (BBB) poorly, demonstrated by the lack of vascular effects on cerebral arteries in the study of Tvedskov et al. [45] but for MK-8825 such data are not available. The issue could possibly be solved by using CGRP(8–37) as a CGRP receptor antagonist, which as a peptide should not pass the BBB.

### Mechanism of CGRP receptor inhibition

If CGRP is required for the fast nociceptive processes induced by nitroglycerin but not for the delayed headaches, what is then the mechanism of CGRP receptor inhibition preventing nitroglycerin-induced increase in spinal trigeminal activity? CGRP is likely to operate as co-transmitter in the spinal trigeminal nucleus [51] and may be particularly important for initiation of central sensitisation [52]. In animals hypersensitive to CGRP due to over-expression of the CGRP receptor protein RAMP1, CGRP has been found to induce mechanical allodynia [53], most likely a central effect. Neuronal structures on the level of the first synapse are certainly central terminals of primary afferents, since CGRP receptor expression has been found in superficial laminae but not in second order neurons in the spinal trigeminal nucleus [54]. Fast sGC-induced processes, as discussed above, may facilitate nociceptive transmission. The delayed transcriptional processes induced by nitroglycerin include probably the upregulation of NO-producing neurons [31]. NO released by these neurons may operate as retrograde transmitter facilitating neurotransmitter release from primary trigeminal terminals, similar to neurons in the spinal dorsal horn, for which an analog function has been postulated [55]. Central sensitisation accompanying migraine attacks, typically diagnosed by cutaneous allodynia of trigeminal areas, may be the result of such long-term changes [56,57]. Triptans are no longer fully effective for migraine therapy after central sensitisation has developed [58].

The established role of CGRP as a key mediator in migraine is underlined by a recent report on elevated CGRP plasma levels during the interictal phases in chronic migraine [59]. In this increasingly prevalent disease a preventive therapy targeting CGRP may be even more important, particularly if the pain is refractory to conventional therapies. Thus it is encouraging that, among others, several approaches to block CGRP or CGRP receptors are in an experimental state, including antibodies and mirror-image RNAs (so-called Spiegelmers) binding to CGRP or CGRP receptors as well as new CGRP receptor antagonists like those discussed in the present paper [60]–[62]. To guide these developments, a better understanding of the events linked to CGRP release and to CGRP actions is desirable, which is the intention of the present and further preclinical studies.

## Conclusions

In the present experiments inhibition of CGRP receptors prevented the increase in spinal trigeminal activity induced by infusion of nitroglycerin, later followed by a decrease in activity below baseline. We assume that CGRP receptor inhibition operates preferably in the spinal trigeminal nucleus to block neuronal activity induced by activation of intracellular NO receptors (soluble guanylate cyclase) at an early state. Assuming that similar mechanisms are involved in the development of spontaneous headache attacks, CGRP receptor inhibition may be also preventive in migraine.

## Competing interests

The authors declare to have no competing interests.

## Authors’ contributions

SF and SA performed the experiments. SF and KM analysed the data and drafted the manuscript. KM instructed the experiments and revised the manuscript. All authors read and approved the final manuscript.
